# Clinical Validation of Deep Learning for Segmentation of Multiple Dental Features in Periapical Radiographs

**DOI:** 10.3390/bioengineering11101001

**Published:** 2024-10-05

**Authors:** Rohan Jagtap, Yalamanchili Samata, Amisha Parekh, Pedro Tretto, Michael D. Roach, Saranu Sethumanjusha, Chennupati Tejaswi, Prashant Jaju, Alan Friedel, Michelle Briner Garrido, Maxine Feinberg, Mini Suri

**Affiliations:** 1 Division of Oral & Maxillofacial Radiology, Department of Care Planning & Restorative Sciences, School of Dentistry, University of Mississippi Medical Center, 2500 North State Street, Jackson, MS 39216, USA; 2 Department of Oral Medicine and Radiology, SIBAR Institute of Dental Sciences, Guntur 522509, Andhra Pradesh, India; 3 Department of Biomedical Materials Science, School of Dentistry, University of Mississippi Medical Center, 2500 North State Street, Jackson, MS 39216, USA; 4 Department of Oral Surgery, Regional Integrated University of Alto Uruguai and Missions, Erechim 99709-910, Brazil; 5 Department of Oral Medicine and Radiology, Rishiraj College of Dental Sciences & Research Centre, Bhopal 462036, Madhya Pradesh, India; 6 VELMENI Inc., 333 W Maude Ave, Sunnyvale, CA 94085, USA; 7 Department of Oral Pathology, Radiology and Medicine, Kansas City School of Dentistry, University of Missouri, 650 East 25th Street, Kansas City, MO 64108, USA

**Keywords:** artificial intelligence, deep learning, periapical radiographs, dentistry, diagnosis, caries, implants, fixed prosthesis, dental restoration, teeth numbering

## Abstract

Periapical radiographs are routinely used in dental practice for diagnosis and treatment planning purposes. However, they often suffer from artifacts, distortions, and superimpositions, which can lead to potential misinterpretations. Thus, an automated detection system is required to overcome these challenges. Artificial intelligence (AI) has been revolutionizing various fields, including medicine and dentistry, by facilitating the development of intelligent systems that can aid in performing complex tasks such as diagnosis and treatment planning. The purpose of the present study was to verify the diagnostic performance of an AI system for the automatic detection of teeth, caries, implants, restorations, and fixed prosthesis on periapical radiographs. A dataset comprising 1000 periapical radiographs collected from 500 adult patients was analyzed by an AI system and compared with annotations provided by two oral and maxillofacial radiologists. A strong correlation (R > 0.5) was observed between AI perception and observers 1 and 2 in carious teeth (0.7–0.73), implants (0.97–0.98), restored teeth (0.85–0.89), teeth with fixed prosthesis (0.92–0.94), and missing teeth (0.82–0.85). The automatic detection by the AI system was comparable to the oral radiologists and may be useful for automatic identification in periapical radiographs.

## 1. Introduction

Radiographs play an essential role in the diagnosis, management, and treatment planning of dental diseases [1,2,3,4]. Periapical radiographs (PA) help diagnose dental caries, periapical lesions, root fractures, and other dental pathologies by allowing detailed visualization of the teeth and surrounding anatomical structures [2,3]. PA radiographs offer several advantages, including high resolution, providing detailed images of the objects under study, being performed in a focused area to avoid overlaps, exposing patients to a low dose of radiation, and demonstrating good cost-effectiveness. Consequently, they are routinely employed in dental practice. However, these radiographs often suffer from image distortions, artifacts, and superimpositions, leading to potential misinterpretation [3,5]. An automatic detection method for periapical radiograph evaluation is essential to overcome these challenges and improve diagnostic accuracy [1]. 

Artificial intelligence (AI) is a branch of computer science that is dedicated to developing intelligent computer systems capable of effectively performing complex human cognitive functions such as problem solving, speech recognition, decision making, learning, planning, and understanding human behavior [1,6,7]. AI can be classified into weak AI, which performs specific tasks using trained programs and includes technologies like natural language processing (e.g., GPT-3—OpenAI) and computer vision (e.g., Face ID—Apple). It can also be classified into strong AI, which aims to achieve human-level intelligence and consciousness, offering multitasking and flexibility, raising ethical concerns and discussions [4]. Since its conception in 1956, AI has found numerous applications across diverse fields, including medicine [8,9,10]. AI has immense potential in medicine, from automated disease diagnosis to facilitating complex procedures such as assisted surgeries. AI applications in dentistry span various specialties, including radiology, endodontics, periodontics, oral and maxillofacial surgery, and orthodontics [4,11,12,13,14,15]. Previous studies have demonstrated that AI has significant potential to improve dental disease diagnosis and treatment planning, thereby reducing errors in dental practice. To achieve these results, the amount of data analyzed during AI training will play a crucial role. Generally, the greater the amount of data analyzed, the better the outcomes [1].

Machine learning (ML) is a subset of AI that enables computer models to make independent predictions by learning patterns from datasets which can be classified into supervised, semi-supervised, and unsupervised learning based on the theory of methods [4,16]. ML systems can further refine prediction accuracy by using algorithms that analyze and undergo training on datasets, thereby enhancing automated learning capabilities [11,17,18]. However, traditional ML techniques involve significant human intervention, increasing their susceptibility to errors and inefficiencies [17,19]. Thus, a more autonomous, multi-layered neural network system called deep learning (DL) was developed to address these limitations [2,11,12,17,19].

DL is a specialized branch of ML comprising computational models capable of learning hierarchical features from data beyond mere pattern recognition, enabling the detection of complex structures such as lines, edges, textures, shapes, and even detailed anatomical features [13,17]. A basic deep learning model consists of at least three layers [4]. DL models consist of interconnected and sequentially stacked processing units called neurons, which form artificial neural networks (ANNs) [17,19]. These ANNs comprise an input layer, which receives raw data and feeds them into the network; multiple hidden layers, which perform various transformations and feature extractions from the input data; and an output layer, which generates the final output or prediction based on the processed information from the hidden layers [11,13]. This enables ANNs to mimic the human brain’s analytical processing and exhibit superior information-processing and learning capabilities. ANNs that can process digital signals like sound, images, and videos through convolutional mathematical operations are called convolutional neural networks (CNNs) [17]. They use a sliding window approach to detect, segment, and classify intricate patterns [20]. This enables automatic feature detection in two-dimensional (2D) and three-dimensional (3D) images. CNNs differ from ANNs primarily in their architecture. CNNs are designed with convolutional layers that apply filters to input data, generating feature maps and reducing image complexity through weight sharing. They also include pooling layers that decrease the dimensionality of these feature maps, enabling more efficient feature extraction. After convolution and pooling, CNNs use fully connected layers to transform 2D feature maps into 1D vectors for classification. This specialized structure allows CNNs to achieve higher efficiency and accuracy in image recognition compared to traditional ANNs [4]. VELMENI Inc. (Sunnyvale, CA, USA) developed a specialized CNN architecture tailored for dental AI applications. This architecture excels at detecting dental findings in periapical radiographs. This innovative CNN model leverages deep learning to enhance precision in dental image analysis, advancing diagnostic capabilities.

The present study aims to evaluate the diagnostic performance of VELMENI Inc.’s AI system in automatically detecting teeth, caries, implants, restorations, and other dental features on periapical radiographs. By harnessing advanced AI techniques, this study seeks to enhance the accuracy, efficiency, and reliability of periapical radiograph interpretations, ultimately contributing to improved dental care and patient outcomes.

## 2. Materials and Methods

Radiographic dataset: One thousand anonymized periapical radiographs taken between June 2022 and May 2023, from individuals aged 18 and older, were obtained from the EPIC and MiPACs systems of the Department of Oral and Maxillofacial Radiology at the University of Mississippi Medical Center. Out of the total of 1000 periapical radiographs, only 500 were used to identify teeth, caries, implants, restorations, and fixed prostheses. Only de-identified periapical radiographs with at least 16 teeth and findings of caries, implants, restorations, periapical pathology, and fixed prostheses were included in the study. Periapical radiographs with no visible teeth were excluded. Periapical and bitewing radiographs with artifacts caused by patient position, beam angulation, patient motion, overlap, or the superposition of foreign objects were not included in this study. All periapical radiographs were obtained using XDR sensors (Los Angeles, CA, USA), set with parameters of 70 kVp, 8 mA, and 0.16 s. The research protocol was approved by the IRB (2024-146).

Convolutional neural networks (CNNs): CNNs have transformed image recognition by effectively handling complex and large inputs. Unlike traditional neural networks, CNNs excel in detecting local features, such as edges and shapes, through convolution operations using filters or kernels. This ability enables CNNs to recognize patterns within images, making them particularly adept at identifying objects. Deep CNNs with multiple layers maintain high accuracy even when objects in images shift or change shape, thanks to their compositional structure. CNNs are powerful tools widely used in dental AI applications for image analysis. 

Image annotation: Five hundred anonymized periapical dental radiographs were analyzed and labeled by two independent oral and maxillofacial radiologists, each with a minimum of five years of experience. The two radiologists underwent the same calibration process and had comparable years of experience prior to participating in the study. The specialists independently identified and detected teeth, caries (including all types such as enamel, dentin, secondary, root, etc.), implants, restorations (amalgam and composites), and fixed prostheses. The radiographs were labeled to highlight the number of teeth with caries, the number of implants, the number of teeth with fillings, the number of teeth with fixed dental prostheses (FDPs), and the number of missing teeth. In addition to the human analysis, a convolutional neural network (CNN) architecture developed by VELMENI Inc., located in California, USA, was utilized to detect the same dental categories in the periapical radiographs. The performance of the artificial intelligence (AI) system was compared by the two radiologists. The agreement between the AI system and observer 1 was indicated by a filled black circle, while the agreement between the AI system and observer 2 was indicated by an empty red circle. This comparative approach aimed to evaluate the efficacy of AI in detecting dental anomalies and to verify the consistency between human and automated assessments.

Statistical analysis: The Pearson product–moment correlation coefficient, commonly known as the Pearson product–moment, is a statistical tool that assesses how strongly and in what direction two continuous variables are linearly related. It is a measure of the linear association between two variables, where a value of r = 1 indicates a perfect positive correlation, and a value of r = −1 indicates a perfect negative correlation. This coefficient is the most widely used method for evaluating linear relationships. Pearson’s product–moment correlation coefficient was used to compare the observations between AI-detected dental findings and observers 1 and 2.

## 3. Results and Interpretation

**Figure 1:**
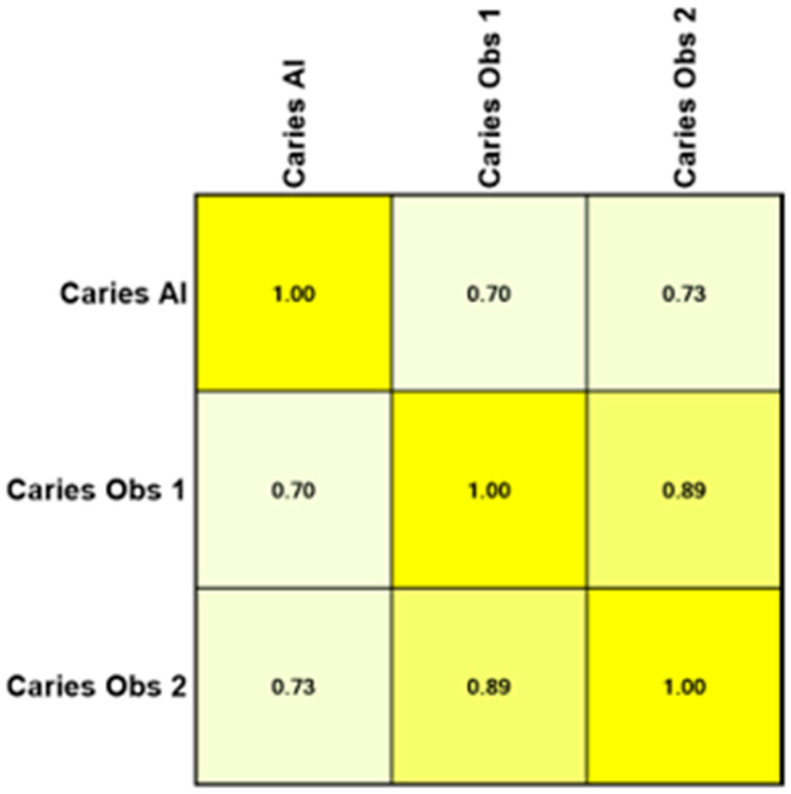

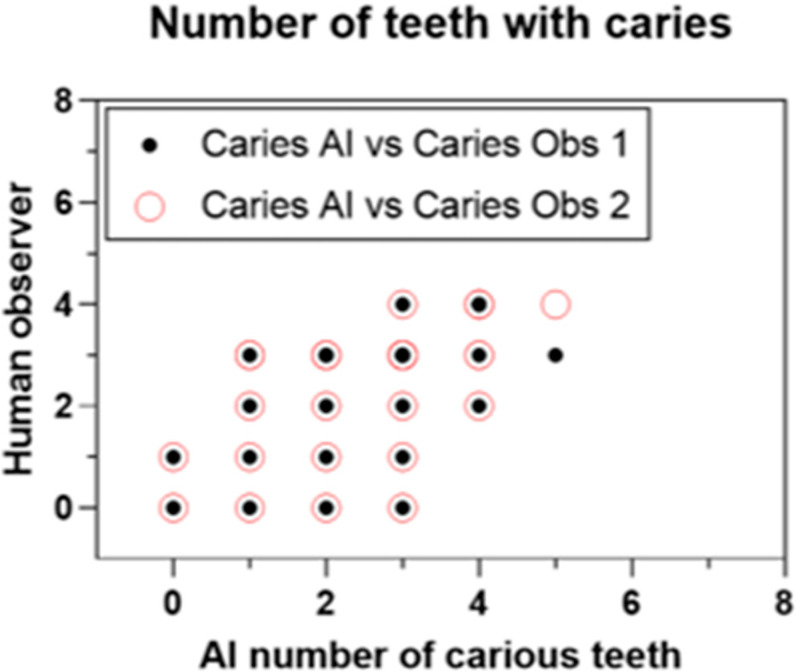
Interobserver agreement (Pearson product–moment) for the number of teeth with caries.

**Figure 2:**
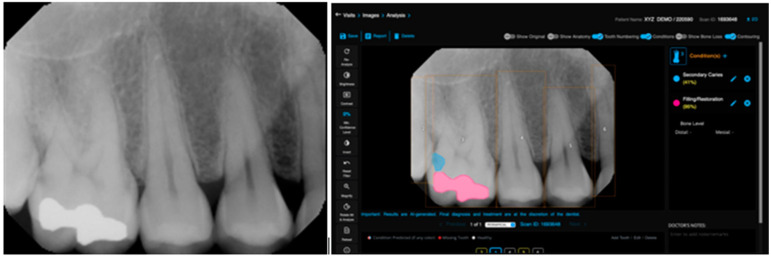
Teeth numbering, caries, and restoration detection on a periapical radiograph using the deep learning algorithm.

**Figure 3:**
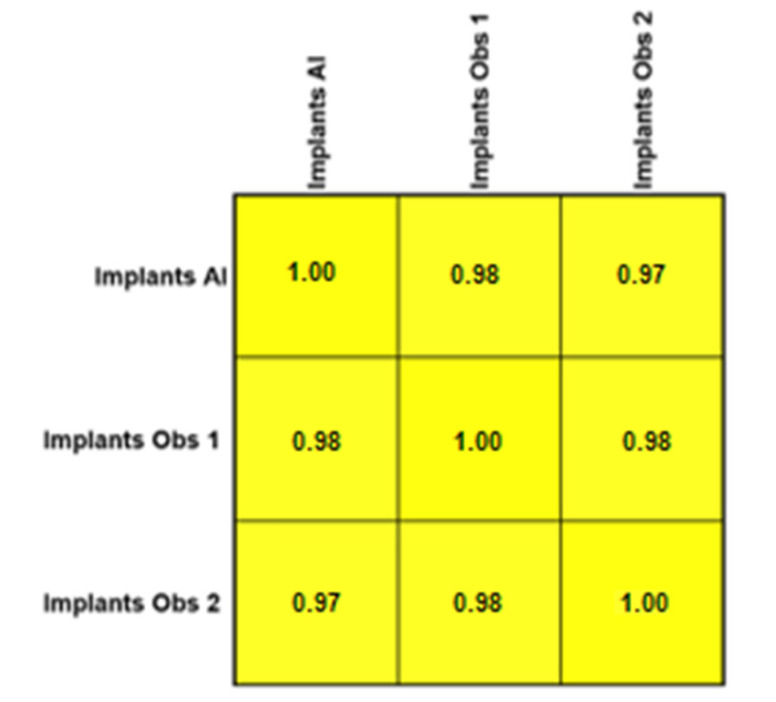

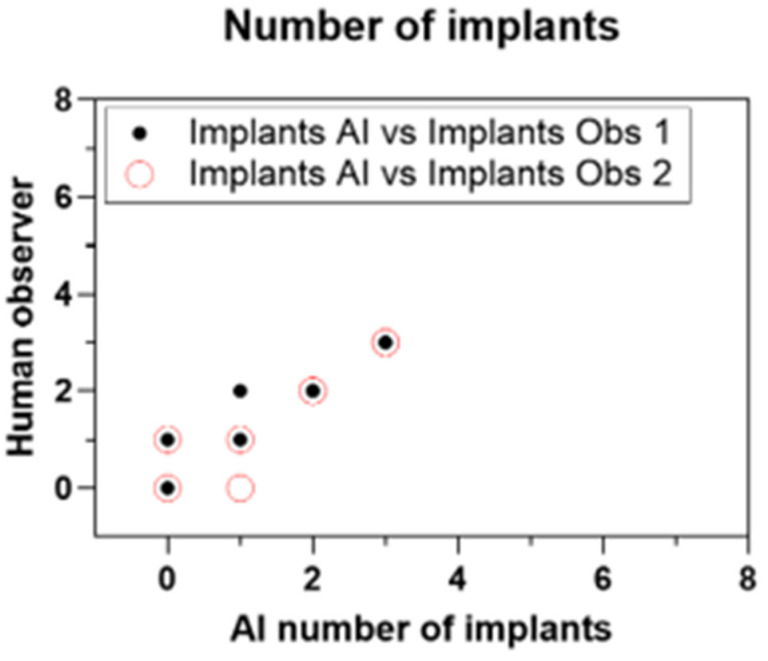
Interobserver agreement (Pearson product–moment) for the number of implants.

**Figure 4:**
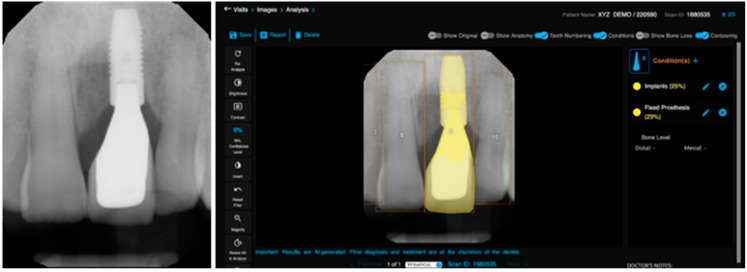
The automatic feature segmentation of the teeth numbering, implant, and fixed prosthesis detection in an anterior periapical radiograph.

**Figure 5:**
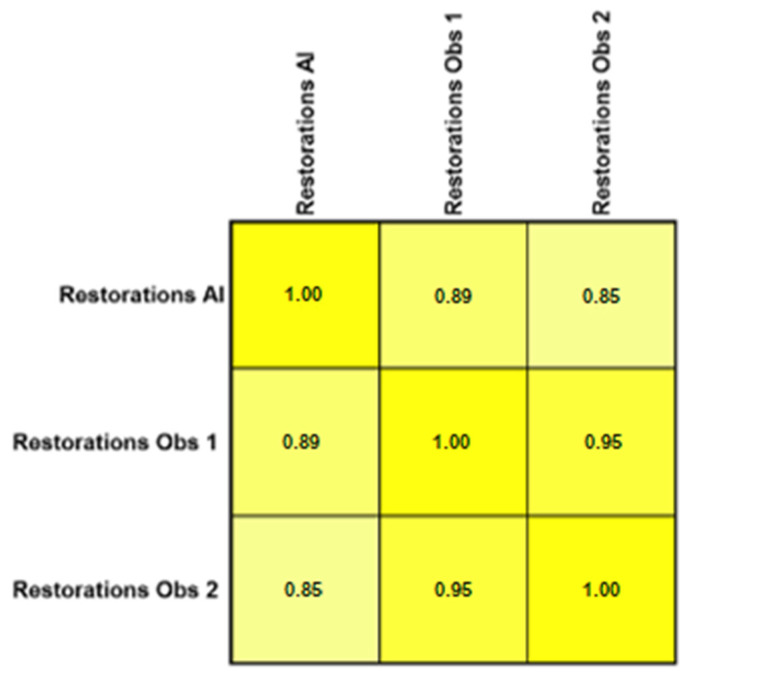

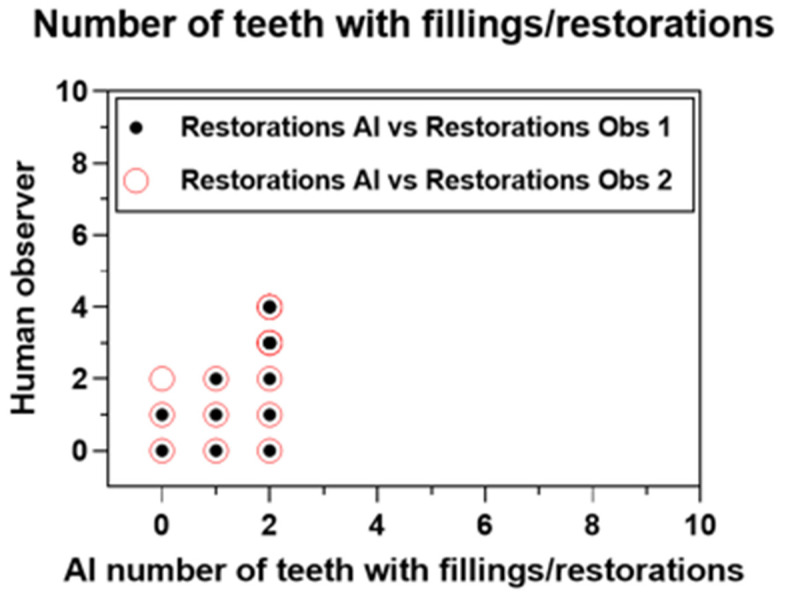
Interobserver agreement (Pearson product–moment) for the number of teeth with fillings.

**Figure 6:**
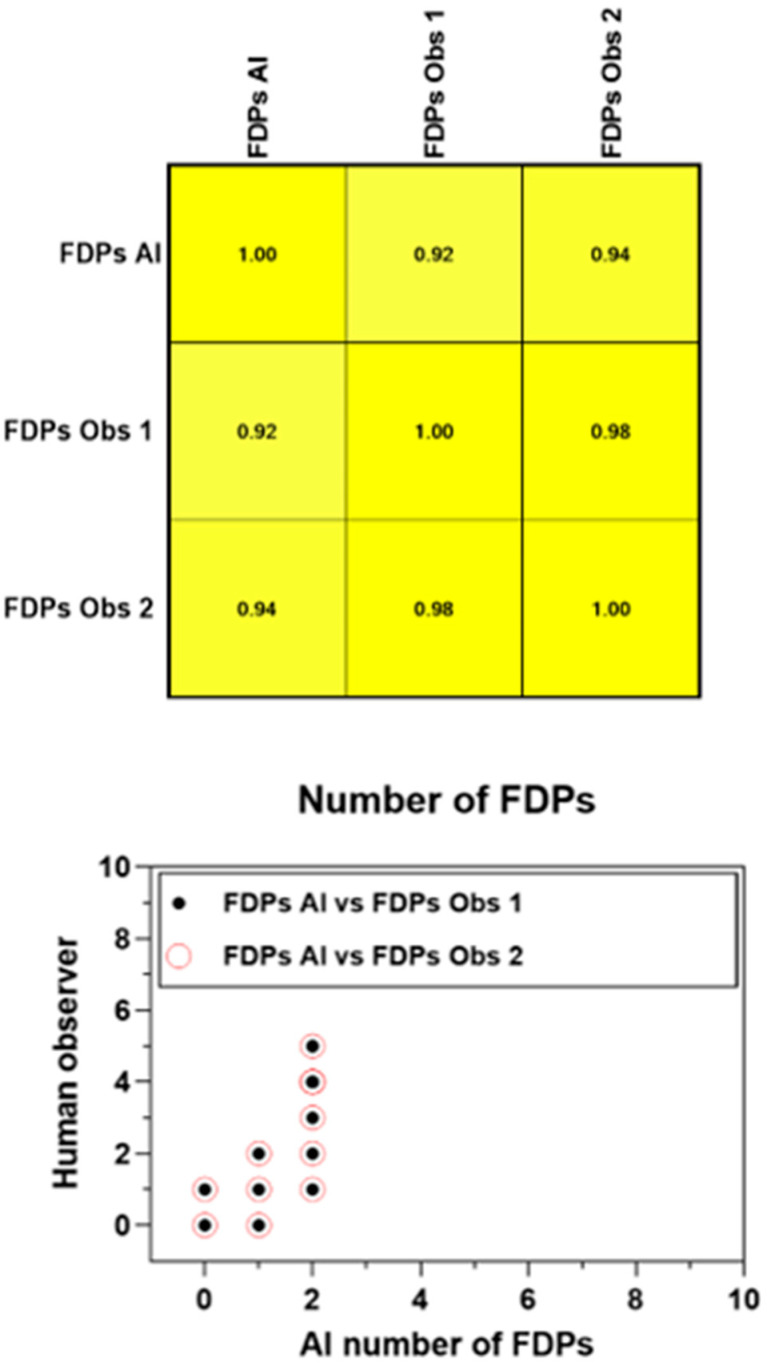
Interobserver agreement (Pearson product–moment) for the number of teeth with FDPs.

**Figure 7:**
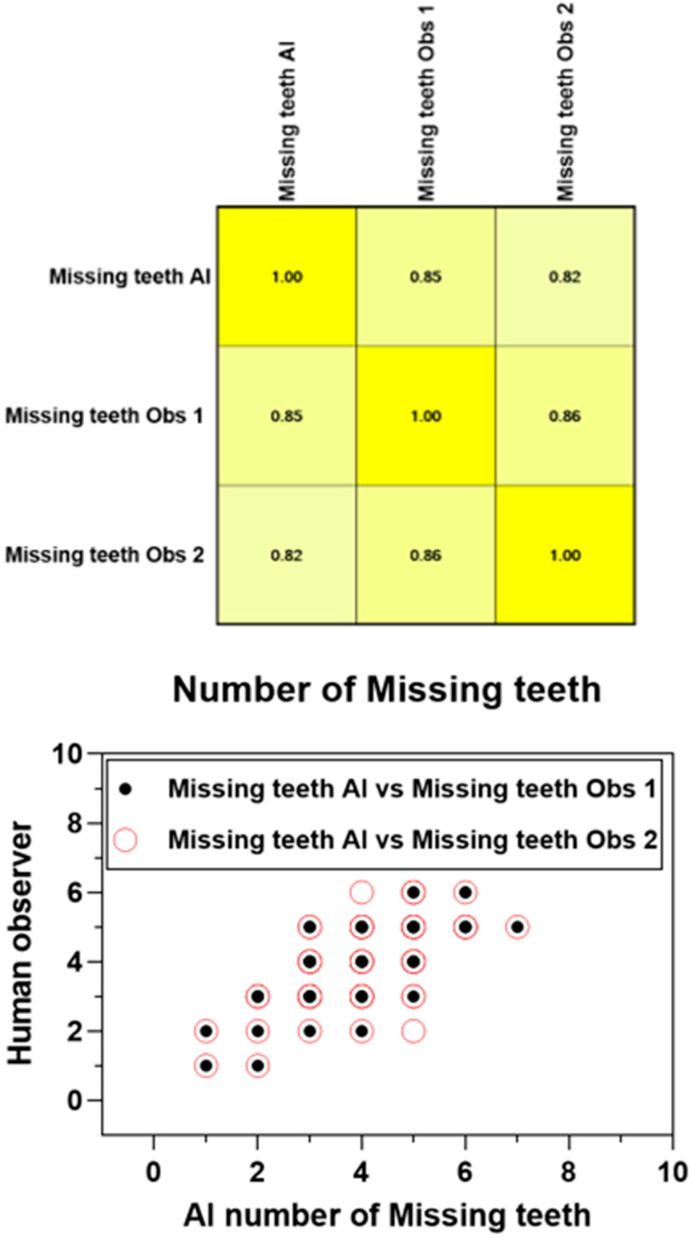
Interobserver agreement (Pearson product–moment) for the number of missing teeth.

The Pearson product–moment showed a strong correlation (R > 0.5) between the perception of the AI and the perceptions of observer 1 and observer 2 for all structures that were identified in the periapical radiograph. For the number of teeth with caries, the AI correlation was found to be 0.70–0.73 (Figure 1 and Figure 2). This demonstrates the AI’s capability to accurately detect caries, which is critical for early diagnosis and intervention. For the number of implants, the AI correlation was found to be 0.97–0.98 (Figure 3 and Figure 4). This near-perfect agreement showcases the AI system’s proficiency in identifying implants, which is vital for patient treatment planning and follow-up. For the number of teeth with fillings, the AI correlation was found to be 0.85–0.89 (Figure 2 and Figure 5). This strong correlation, reflecting the AI’s accuracy in detecting dental restorations, is essential for assessing the integrity and longevity of dental restorative treatments. For the number of teeth with fixed prostheses, the AI correlation was found to be 0.92–0.94 (Figure 4 and Figure 6), while that for the number of missing teeth was found to be 0.82–0.85 (Figure 7). These capabilities of the AI system are crucial for comprehensive dental evaluations and indicate a high level of reliability in identifying edentulous areas, which is important for treatment planning and prosthetic considerations.

## 4. Discussion

Accurate diagnosis is a cornerstone of effective dental practice, necessitating the use of reliable diagnostic tools. Among these, periapical radiography stands out due to its ability to provide detailed images of the teeth and their supporting bone. Its widespread use is attributed to several advantages: a high spatial resolution, a straightforward technique, a minimal radiation dose being administered to the patient, cost-effectiveness, and a painless process for the patient [3]. However, periapical radiography is not without its limitations. Issues such as the presence of artifacts inherent to its technique and limited visualization of surrounding structures can sometimes lead to incorrect interpretations by clinicians. To address these challenges, the adoption of automatic detection methods to assist dental professionals in interpreting periapical radiographs is becoming increasingly important. These advanced methods enhance diagnostic accuracy and improve treatment planning, ultimately benefiting patient care and limiting human error.

This study aimed to assess the diagnostic capabilities of VELMENI Inc.’s automated system in identifying teeth, caries, implants, restorations, and fixed prostheses on periapical radiographs. Using Pearson product–moment correlation coefficient analysis, the results underscored the level of agreement between the VELMENI AI system, which utilizes CNN architecture, and the interpretations of two expert human observers.

Convolutional neural networks (CNNs) are extensively utilized and have shown outstanding performance in tasks such as image segmentation and teeth detection [21,22]. Tuzoff et al. studied an automated system for tooth detection and numbering, discovering that the CNN’s performance matched that of experts, and could potentially save clinicians time through automated dental charting. However, they also emphasized that this process would assist dentists in their decision-making process rather than substitute them [23]. Similar results were obtained in our study, where we found a high correlation (R = 0.85 and 0.82) between the AI dataset and the observers 1 and 2, respectively, for the recognition of missing teeth.

CNNs have been primarily employed to create AI-based systems for caries detection, as they can take images as inputs and produce image identification and classification as outputs. A systematic review reported that the accuracy of AI-based models in predicting caries ranges from 83.6% to 97.1% across various studies. Moreover, AI support for dentists in detecting enamel caries led to a substantial improvement, with detection rates increasing from 44.3% to 75.8% [21,24]. The present study also found a strong correlation in caries detection between the AI and the two human observers, with Pearson correlation coefficients of R = 0.7 with observer 1 and R = 0.73 with observer 2. It is important to note that early caries detection is fundamental for preserving tooth tissues, delaying restorative interventions, reducing expensive restorative treatments, and retaining teeth long-term, and can be sometimes missed by inexperienced clinicians [24,25]. In a study performed by Garcia Cantu et al., it was concluded that the trained neural network outperformed most dentists in accurately detecting caries lesions on bitewings, particularly for initial lesions. Moreover, while dentists tended to under-detect lesions, the neural network slightly over-detected them [26]. However, they compared the efficiency of AI against experienced general dentists, rather than oral and maxillofacial radiologists, who are the experts in interpreting these radiographs, as was performed in our study.

The application of convolutional neural networks for identifying dental implants is a well-explored area within AI and implantology, where multiple studies have shown promising results in detecting dental implants in both panoramic and periapical radiographs [27]. These outcomes are consistent with our findings, where the AI demonstrated the highest agreement with the two human observers in identifying the number of dental implants (R = 0.97–0.98). These results could offer valuable assistance to dentists in identifying implants in instances when the patient does not recall having a dental implant placed, or its location within the oral cavity. Dental implants have become increasingly popular, as it is a permanent way to restore function after losing a tooth. Along with this trend, more types and options have become available in the market, most of them with different shapes, sizes, and overall architectures. By using deep learning algorithms, it is now possible to determine not only its presence, but also identify the implant’s brand [28,29]. However, the studies published in the scientific literature often used a limited number of brands, leaving out many systems available on the market, mainly due to the large number of radiographs needed to train the machine, making this task extremely difficult to achieve.

Diagnosing various types of restorations might be straightforward for expert clinicians but challenging for less experienced dentists. Detecting tooth-colored restorations through visual inspection can be difficult at times, regardless of a clinician’s expertise. A study employing a CNN achieved promising results in detecting metallic restorations, with a sensitivity of 85.48%, whereas the sensitivity for detecting composite resin restorations was 41.11% in panoramic radiographs. However, they observed that the AI-based software showed higher detection errors for resin-based restorations in cases where even the operators had difficulty identifying them [30]. A study conducted by Abdalla-Aslan et al. found 100% and 83.1% sensitivity to detect amalgam and composite fillings, respectively, using AI in panoramic radiographs [31]. It is important to consider that periapical radiographs have higher spatial resolution compared to panoramic radiographs, making restoration detection and recognition more reliable. The present study found a strong correlation between the CNN and the two human observers, with correlation coefficients ranging from R = 0.85 to R = 0.89. These results underscore the potential for using CNNs in diagnosing and planning treatments for patients, educating students, and practising forensic dentistry.

The detection of the number of teeth with fixed dental prostheses (FDPs) yielded the second-best results along with the identification of the number of missing teeth mentioned earlier. In our study, a strong correlation was found between observers 1 and 2 and the AI, being R = 0.92 and R = 0.94, respectively. On the contrary, a study conducted by Choi et al. obtained a low performance when detecting prostheses, mainly due to the wide variety of shapes, the radiopacity, and the materials used for prosthesis. It is also important to note that they used panoramic radiographs, which are indeed more complex to interpret when trying to detect different materials [32].

The results of this study highlight the potential of AI systems based on deep learning for automatically detecting teeth, caries, implants, restorations, and fixed prostheses on periapical radiographs. These systems can enhance diagnostic efficiency, accuracy, and consistency, while reducing repetitive tasks. Additionally, machine learning in dentistry can support fundamental dental radiography, complement further clinical exams, aid in training future dental practitioners, and assist in forensic body identification. Despite the promising outcomes in identifying common conditions on periapical radiographs, the study’s limitations must be acknowledged. The dataset used was limited and lacked external data, which may affect the generalizability of the results. To overcome these limitations, future research should utilize larger and more diverse datasets to provide deeper insights and strengthen the validity of the findings.

Even with the recent advancements in AI technology for detecting dental lesions, the novelty and practical application of this technology in dental radiology remain areas of ongoing development. To improve accuracy in diagnosing caries and periodontal disease, we highlight the importance of integrating advanced machine learning algorithms with extensive and diverse datasets. The cost of these services may be variable and will depend on demand for the software. We suggest exploring partnerships with technology providers and leveraging economies of scale through collaborative research, which may lead to more affordable solutions as AI technology becomes more prevalent and cheaper. These measures aim to enhance the reliability and accessibility of AI in clinical practice, addressing key concerns and paving the way for its effective integration into routine dental care.

In conclusion, it has been stated that more experienced examiners show an improved diagnostic accuracy compared with less experienced ones. Yet, any qualified clinician can suffer from both mental and eye fatigue, which could lead to them ignoring important radiographic signs that can interfere with their final diagnostic decisions, resulting in an incorrect or misinterpreted diagnosis. This problem can be avoided by the use of AI, significantly improving diagnostic accuracy and consistency, as it has been proven that it can automatically detect conditions such as caries, periodontal disease, missing teeth, restorations, pathosis, etc., that might be missed by human examiners [30].

Another clinical application of AI is workflow optimization, as it facilitates automated report generation and seamless integration with electronic health records, thus streamlining data and reducing the time clinicians spend on manual tasks. AI also plays a crucial role in education by providing training tools and decision support systems that enhance diagnostic skills and support decision-making, especially in academic institutions. Additionally, AI improves patient communication through visual aids and educational materials that help patients understand their conditions and treatment options better. Finally, AI contributes to research and development by analyzing large datasets to uncover trends and innovations, driving advancements in dental diagnostics and treatment technologies [33]. 

This study showcases the potential of modern CNN architectures for automated dental X-ray interpretation [33,34]. These results confirm that AI, and specifically this approach, is of sufficiently high quality to be incorporated into software for real-world applications and daily practice. Future research is needed to advance the current knowledge and disease recognition from other anatomical areas of the maxillofacial skeleton utilizing conventional and volumetric images, such as cone beam computed tomography. 

## Data Availability

The datasets presented in this article are not readily available because due to privacy or ethical restrictions.

## Abbreviations

AI—artificial intelligence; ML—machine learning; DL—deep learning; ANN—artificial neural network; CNN—convolutional neural network; 2D—two-dimensional; 3D—three-dimensional.
